# A truly green synthesis of α-aminonitriles *via *Strecker reaction

**DOI:** 10.1186/2191-2858-1-11

**Published:** 2011-10-04

**Authors:** Debasish Bandyopadhyay, Juliana M Velazquez, Bimal K Banik

**Affiliations:** 1 Department of Chemistry, The University of Texas-Pan American, 1201, West University Drive, Edinburg, TX 78539, USA

## Abstract

**Background:**

The classical Strecker reaction is one of the simplest and most economical methods for the synthesis of racemic α-aminonitriles (precursor of α-amino acids) and pharmacologically useful compounds.

**Results:**

Indium powder in water is shown to act as a very efficient catalyst for one-pot, three-component synthesis of α-aminonitriles from diverse amines, aldehydes and TMSCN. This general rapid method is applicable to a wide range of amines and aldehydes and produces products in excellent yield.

**Conclusions:**

The present one-pot, three-component environmentally benign procedure for the synthesis of α-aminonitriles will find application in the synthesis of complex biologically active molecules.

## Background

Strecker reaction [1], the oldest known synthesis of α-aminonitriles, is one of the most general methods potentially useful for syntheses of amino acids and other bioactive compounds including natural products. In addition, the Strecker reaction represents one of the simplest and most economical methods for the preparation of α-amino acids for both laboratory and industrial scales [2]. Since 1850, a number of publications have appeared on this reaction. Still this reaction is under active investigation. Recently, synthesis of hepatitis C virus NS3 serine protease inhibitors [3], (±)-phthalascidin 622 [4] and novel boron-containing retinoids [5] have been reported following this strategy. A number of new catalysts have also been reported for this reaction which includes mesoporous aluminosilicate (Al-MCM-41) [6], lanthanum(III)-binaphthyl disulfonate [7], nanocrystalline magnesium oxide [8], BINOL-phosphoric acid [9,10], Fe(Cp)_2_PF_6 _[11], Jacobsen's thiourea catalyst [12], *N*-heterocyclic carbene (NHC)-amidate palladium(II) complex [13], Yb(OTf)_3_-pybox [14], K_2_PdCl_4 _[15], gallium (III) triflate [16], bisformamides [17], IBX/TBAB [18], Lewis base e. g. N,N-dimethylcyclohexylamine [19], superparamagnetic iron oxide [20], and ionic liquid [21]. To prepare α-aminonitriles (precursor to α-amino acids) generally an imine is reacted with a cyanide source. Notable among them are HCN [22], KCN [23], (EtO)_2_P(O)CN [24,25], Et_2_AlCN [26,27], Bu_3_SnCN [28,29], and TMSCN [3,4,6-20]. Among these cyanide sources, trimethylsilyl cyanide (TMSCN) is relatively easy to handle and highly soluble in organic solvents. In contrast, many of these reported methods involve the use of expensive reagents, hazardous solvents, longer reaction times and tedious workup procedure. Therefore, it is desirable to develop an efficient and practical method for the Strecker reaction under eco-friendly conditions.

## Results

**Figure 1:**
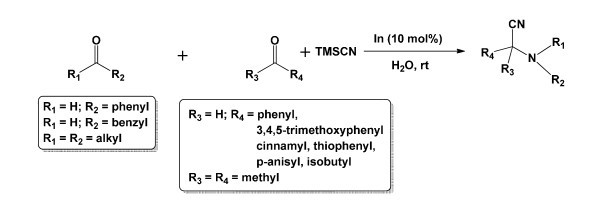
**Three component Strecker reaction using amines (1 mmol), carbonyl compounds (1 mmol) and TMSCN (1.2 mmol) in water in presence of indium (10 mol%)**.

We have been working on the synthesis and biological evaluation of various β-lactams as novel anticancer agents [30-35] over the past several years. The synthesis of β-lactams through imines requires a carbonyl compound and an amine. Our study suggests that carbonyl compounds, amines and TMSCN in the presence of a mild acidic reagent will lead to the synthesis of α-aminonitriles in good to excellent yield. This hypothesis has been tested by reacting several amines with various carbonyl compounds and TMSCN in the presence of indium as catalyst. Recently, organic reactions in water have received much attention in view of green methodologies [36]. First of all, indium and a number of indium salts have been screened using aniline, benzaldehyde and TMSCN as a model reaction at room temperature. The results are shown in Table 1. The reaction was then performed in various solvents using indium as the catalyst to identify the best condition. It suggests that indium is the best catalyst in aqueous medium for the reaction (Table 2). The same reaction was used to optimize the amount of the catalyst. The results show (Table 3) that 10 mol% indium is required to complete the reaction in 30 minutes. Considering the above observations we carried out a series of reaction using various carbonyl compounds, amines and TMSCN in presence of indium (10 mol%) in water as solvent (Figure 1). In all the cases, the reactions were completed within 30 min to 1.5 hr and the products were obtained in excellent yield (Table 4). The products have demonstrated satisfactory spectral and mp data with the reported values.

**Table 1:** Three component Strecker reaction using aniline (1 mmol), benzaldehyde (1 mmol) and TMSCN (1.2 mmol) in water (30 min): catalyst optimization

Entry	Catalyst (10 mol %)	Yield (%)^a^
1	Indium	98

2	Indium (II) chloride	70

3	Indium (III) chloride	82

4	Indium (III) bromide	85

5	Indium selenide	62

6	Indium oxide	48

^a^isolated yield

**Table 2:** Three component Strecker reaction using aniline (1 mmol), benzaldehyde (1 mmol) and TMSCN (1.2 mmol) in presence of indium (10 mol%) in various solvents (30 min): solvent optimization

Entry	Solvent	Yield (%)^a^
1	Water	98

2	THF	34

3	Ethanol	56

4	Toluene	60

5	Methanol	68

6	Dichloromethane	61

7	DMSO	76

8	THF/H_2_O (1:1)	54

9	Ethanol/H_2_O (1:1)	71

^a^isolated yield

**Table 3:** Three component Strecker reaction using aniline (1 mmol), benzaldehyde (1 mmol) and TMSCN (1.2 mmol) in water (30 min): optimization of the amount of the catalyst

Entry	Indium (mol %)	Yield (%)^a^
1	30	89

2	25	91

3	20	88

4	15	89

5	10	98

6	5	67

7	2	54

8	1	43

^a^isolated yield

**Table 4:** Three component Strecker reaction using amines (1 mmol), carbonyl compounds (1 mmol) and TMSCN (1.2 mmol) in water in presence of indium (10 mol%)

Entry	Amine	Carbonyl compound	Product	Time (min)	Yield (%)^a^	**Ref.**
1				30	98	[11]

2				75	93	[15]

3				75	79	[11]

4				45	86	[11]

5				60	88	[11]

6				75	91	[11]

7				90	94	[21]

8				75	95	[21]

9				90	97	[21]

10				45	98	[10]

^a^isolated yield

## Discussion

A series of α-aminonitriles were synthesized by using diverse amines, aldehydes and TMSCN in the presence of indium metal (10 mol%) as catalyst in water. As shown in Table 4, the reaction proceeded equally well irrespective of the nature of the carbonyl compounds (aliphatic, aromatic, heteroaromatic) or amines (aliphatic, heterocyclic, and aromatic) to afford the corresponding products in excellent yield (79-98%). The catalytic system worked well with acid sensitive heteroaromatic aldehyde (entries 4, 6, 7), α, β unsaturated aldehyde (entry 3), aliphatic aldehyde (entry 5) and ketone (entry 10). Aromatic primary amine (aniline), benzyl amine (entry 6), heterocyclic amines (entries 7, 8 and 9) could effectively undergo Strecker reaction with aldehydes and TMSCN to give the corresponding products in excellent yields (94-97%). For aliphatic amines such as benzyl amine, piperidine and morpholine relatively slower reaction rate was observed.

A plausible mechanism may follow a two-step pathway. In the first step, indium acts as an Lewis acid to facilitate formation of the corresponding imine from the condensation of the amine and aldehyde. In the subsequent step, the imine is further activated due to the presence of indium, to form a more electrophilic C = N intermediate. As a result, an attack of TMSCN to the imine carbon can take place and thus the corresponding α-aminonitriles is formed via hydrolysis in water.

## Conclusions

There is growing interest in the one-pot Strecker synthesis of α-aminonitriles from carbonyl compounds, amines and TMSCN, because of the significant importance of α-aminonitriles in preparing a wide variety of amino acids, amides, diamines, and nitrogen containing heterocycles. In summary, we have developed a rapid, convenient and efficient one-pot, three-component environmentally benign Strecker reaction using indium as catalyst at room temperature. A series of α-aminonitriles were obtained in excellent yields. This reaction will be applicable to the synthesis of various organic compounds of medicinal interests.

## Methods

### General

FT-IR spectra were registered on a Bruker IFS 55 Equinox FTIR spectrophotometer as KBr discs. ^1^H-NMR (600 MHz) and ^13^C-NMR (125 MHz) spectra were obtained at room temperature with Bruker-600 equipment using TMS as internal standard and CDCl_3 _as solvent. Analytical grade chemicals (Sigma-Aldrich Corporation) were used throughout the project. Deionized water was used for the preparation of all aqueous solutions.

### General procedure for the one-pot, three-component Strecker reaction

A representative experimental procedure (entry 1) is as follows: In powder (11 mg) was added to a mixture of aniline (1 mmol), benzaldehyde (1 mmol) and TMSCN (1.2 mmol) in water (1 mL). The resulting mixture was stirred at room temperature and the progress of the reaction was monitored by TLC. After completion of the reaction (Table 4) diethyl ether was added and the solution was filtered, washed with brine and water. It was dried over anhydrous sodium sulphate and filtered. A short column of silica gel was used to purify the product 2-phenyl-2-(phenylamino)-acetonitrile in 98% yield.

## Competing interests

The authors declare that they have no competing interests.
